# Development of a Chemiresistor Sensor Based on Polymers-Dye Blend for Detection of Ethanol Vapor

**DOI:** 10.3390/s100402812

**Published:** 2010-03-29

**Authors:** Marcos A. L. dos Reis, Fabiano Thomazi, Jordan Del Nero, Lucimara S. Roman

**Affiliations:** 1 Pós-graduação em Engenharia Elétrica, Universidade Federal do Pará, 66075-900, Belém-PA, Brazil; E-Mail: marcosallan@ufpa.br; 2 Departamento de Física, Universidade Federal do Paraná, 81531-990, Curitiba-PR, Brazil; E-Mail: fabiano@fisica.ufpr.br; 3 Departamento de Física, Universidade Federal do Pará, 66075-110, Belém-PA, Brazil

**Keywords:** chemiresistor sensor, PEDOT-PSS, MR dye, ethanol vapor

## Abstract

The conductive blend of the poly (3,4-ethylene dioxythiophene) and polystyrene sulfonated acid (PEDOT-PSS) polymers were doped with Methyl Red (MR) dye in the acid form and were used as the basis for a chemiresistor sensor for detection of ethanol vapor. This Au | Polymers-dye blend | Au device was manufactured by chemical vapor deposition and spin-coating, the first for deposition of the metal electrodes onto a glass substrate, and the second for preparation of the organic thin film forming ∼1.0 mm^2^ of active area. The results obtained are the following: (i) electrical resistance dependence with atmospheres containing ethanol vapor carried by nitrogen gas and humidity; (ii) sensitivity at 1.15 for limit detection of 26.25 ppm analyte and an operating temperature of 25 °C; and (iii) the sensing process is quickly reversible and shows very a low power consumption of 20 μW. The thin film morphology of ∼200 nm thickness was analyzed by Atomic Force Microscopy (AFM), where it was observed to have a peculiarly granulometric surface favorable to adsorption. This work indicates that PEDOT-PSS doped with MR dye to compose blend film shows good performance like resistive sensor.

## 1. Introduction

Chemiresistor sensor is the most common type of configuration of the gas sensor array, because it can be manufactured easily and with low-cost, as well as having many applications [1–6]. This type of sensor is simple, but efficient, and can be made of blend films like an active layer, such as Polypyrrole doped with dodecyl benzene sulfonic acid (DBSA) and ammonium persulfate (APS) and an inkjet-printed PEDOT-PSS layers [7,8], changing electric resistance in the presence of alcohol vapors (propanol, methanol and ethanol). The small alcohol molecule such as ethanol interacts more powerfully in polymeric matrixes than the alcohols with a higher molecular weight, and their high dielectric constant interacts with the nitrogen atoms, decreasing the electric resistance [9]. Moreover, conducting polymers doped with a second component are commonly used compared with functionalized molecular structure of polymers in chemiresistors, the advantage is shun chemical syntheses and improved sensitivity of the sensors. For example, recently Saloducho *et al.* [10] reported a nanostructured gas sensor with improved sensitivity without the need for polymeric modification.

**Figure 1.**
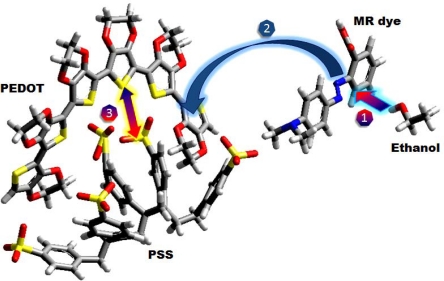
Basic scheme of the chemical reactions, where the analyte is adsorbed by the MR sensitizer (1) The MR improves the interaction between analyte and polymer blends, increasing ionic diffusion; (2) This reduces the Coulomb interaction and enhance the conductivity; (3) The red, yellow, gray, blue and white colors are oxygen, sulfur, carbon and hydrogen atoms, respectively.

Here, PEDOT-PSS was used as a polymeric matrix with the second component MR dye, an azo compound molecule usually used as an acidic/basic indicator and changing color depending on pH environment. This acid molecule has a diimide functional group (*N*=*N*), where the charge recombination occurs [11]. Figure 1 shows that when the ethanol molecule is physisorbed by dipole-dipole interactions on the MR dye molecule, it is admixed into the polymer blend to improve ion diffusion between polymeric interchannels.

In the work reported by Mabrook *et al.* [8], PEDOT-PSS was used without a second component resulting in nonreversible electric currents with a smaller response, *i.e.*, current at 10^−9^ A, to thin organic films. However, a reversible response to sensors with thick films was observed. In both cases, methanol and ethanol vapors were used, but the results are not related to ethanol because poor responses were obtained.

## 2. Experimental Section

### 2.1. Materials

The commercial poly(3,4-ethylene dioxythiophene) doped with polystyrene sulfonated acid (PEDOT-PSS) polymers were obtained in aqueous solution from Bayer Corporation; 2-(4-Dimethylaminophenylazo)benzoic acid, 4-Dimethylaminoazobenzene-2′-carboxylic acid, Acid Red (Methyl Red dye) and Ethanol Alcohol (99.8%) were purchased from Sigma-Aldrich and Vetec, respectively. For gas sensing, nitrogen was used (inert carrier) and ambient air under relative humidity (RH) at 46 % to simulate environment conditions.

## 2.2. Manufacturing gas sensor element

The sensor element was prepared by thermal evaporation and spin-coating. Gold thin film (60 nm) was coated on the glass substrate by thermal evaporation forming electrodes. After that, spin-coating was used to deposit a solution of PEDOT-PSS with MR dye (50%) and gap electrodes of 450 μm at 1,000 rpm forming a organic active area of ∼1.0 mm^2^ with ∼200 nm of thickness to sensing gas. The morphology, as well as thickness was characterized by AFM (SPM-9500 J3, Shimadzu) operating in dynamic mode.

### 2.3. Electrical measurements and gas sensing experiments

The electrical measurements (current-voltage and current-time) of Au | Polymers-dye blend | Au were measured using KEITHLEY 6487 in DC voltage supply. The *I*(*V*) characteristics were obtained under variable temperature in a controlled chamber with 20 mV scanning, but the responses as a function of time was performed in real ambient conditions with nitrogen or/and ethanol flow on the sensor. Moreover, the electric resistance of the sensor element was calculated by Ohm’s Law, resulting in a very low power consumption of 20 μW when operating in air [12].

In general, gas sensing experiments were performed in nitrogen the as gas carrier without or with ethanol vapor *i.e.*, without ethanol to measure the influence of the RH on the sensitivity and with ethanol to obtain responses at analyte. The limit detection of 26.25 ppm of the nitrogen/ethanol mixture can be determined from measured gas-phase concentrations using Henry’s Law [13]. In this case, 5.0 mL of ethanol was used with concentration a in aqueous phase at 5.0 mM and Henry’s Law constant for standard conditions of 1.9 × 10^2^ atm/M, so their evaporation is carried by nitrogen until the surface of the sensor element.

## 3. Results and Discussion

In general, chemiresistors are known by ohmic current-voltage characteristics, *I*(*V*), where the charge transport in the case is explained by space charge-limited current (SCLC). In this model, at low applied voltages the charge is injected through the isolated barriers and disordered conducting polymers without disturbing the equilibrium charge carrier concentration (*n*_0_).

**Figure 2.**
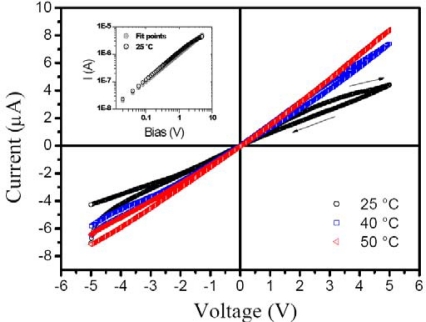
Current-voltage characteristics of a Au | Polymers-dye blend | Au sensor under variable temperature. The measurements were from −5.0 VDC until 5.0 VDC and back at 20 mV of scanning (see arrows). The inset corresponds to log-log curves of the Ohmic equation fitting *I*(*V*) characteristics at room temperature.

The ohmic transport is described by

(1) $$J=e\;\mu \;n_{0}\frac{V}{d}$$

where *J* is the current density, *e* is the elementary charge, *μ* is the charge carrier mobility, *V* is the voltage applied and *d* is polymer film thickness. However, when a high voltage is applied on the electrodes, the current density is proportional at *V*^2^ and expressed by

(2) $$J=\frac{9}{8}\varepsilon \;\mu \frac{V^{2}}{d^{3}}$$

where ε is the permittivity of the polymeric blend. In this case, the charge transport depends on temperature, *i.e.*, phonon-assisted tunneling. Figure 2 shows *I*(*V*) ohmic curves under increasing temperature, so the charge carrier reaches conductivity intermediate levels assisted by phonons and improving the current.

### 3.1. AFM analysis of Polymers-dye Blend Films

**Figure 3.**
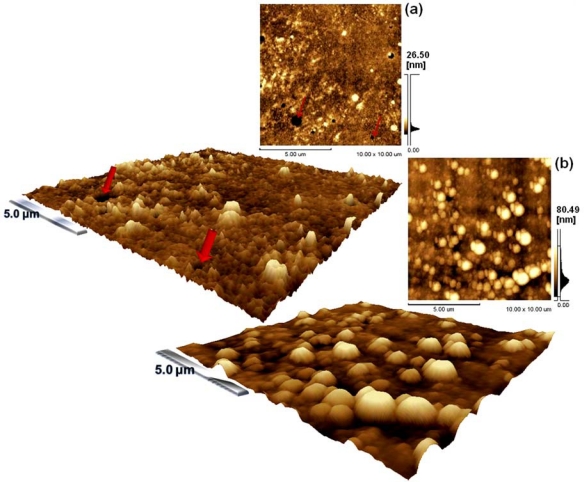
AFM micrographs of PEDOT-PSS doped with MR and deposited at (a) 2,000 rpm or (b) 1,000 rpm. Red arrows indicate defects on the thin film surface in (a). All images are 10 μm × 10 μm of size area.

The morphology was investigated by atomic force microscopy (AFM) of films deposited on glass substrates by spin-coating at 1,000 or 2,000 rpm. After evaporation of the solvent, a thin film shows a granular morphology, with a typical grain size of about 10–80.49 nm at 1,000 rpm. Figure 3a,b shows pictures of topographies - for which the surface area can be observed to decrease with the increasing rotation. The AFM images of the as-deposited films reveal a change on the surface; Figure 3(a) shows topography for Polymers-dye blend deposited at 2,000 rpm with a grain size of 26.50 nm and a surface area of ∼100.55 μm^2^, in comparison with Figure 3(b) with a ∼112.57 μm^2^ surface area when deposited at 1,000 rpm, though both samples were prepared using equal conditions. The higher surface area for chemiresistors is very important to improve the adsorption of analyte enhancing gas sensitivity [14]. In this case, the better sensors manufactured were deposited at 1,000 rpm and obtained ∼200 nm of thickness. Moreover, thin films prepared above this rotation show increased surface defects and small, non-reproducible electric responses.

In addition to nanostructured surfaces *i.e.*, relative to modification of nano-sized grains on thin films, the increase of the surface area by inclusion of nanostructures is a good method. Recently, Vander Wal *et al.* [15] have shown good responses with a nanostructured surface sensor: in this work, nano-sized grains were obtained on the surface by rotation controlled.

### 3.2. Responses as a function of time

**Figure 4.**
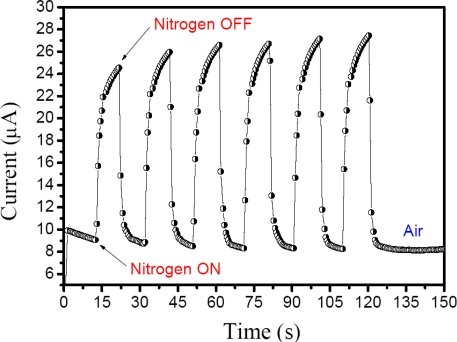
Response of PEDOT-PSS doped with MR upon periodic exposure to nitrogen an applied 2.50 VDC bias in room temperature with 46 % relative humidity (RH).

With respect to the real-time responses of the sensor under nitrogen or analyte vapor, both measurements were obtained quickly and were reversible responses. In Figure 4, the nitrogen supply was turned ***ON*** after 13 s on the thin film surface of the sensor under 46% RH to simulate local ambient conditions, and an immediate increase in electric current was observed. In the next periodic process, the current levels increased slowly. This is due to the fact that water molecules trap electrons and change the ion transfer in the thin film, so that after removal of the water molecules on the film surface, the sensitivity increases while decreasing the resistance [16].

**Figure 5.**
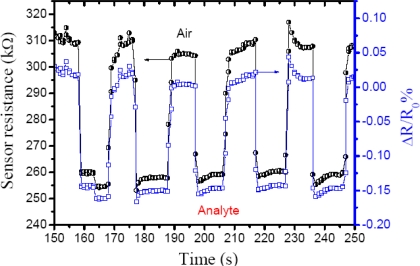
Electric response for Polymers-dye sensor at 26.25 ppm. Ethanol vapor carried with nitrogen gas was turned *ON* after 158 seconds followed by a reproducible process of resistance change from 303.69 kΩ to 264.48 kΩ with normalized resistance decreasing by 0.15 %.

In general, the adsorption of the analyte and water vapors are cooperative [17,18]. Figure 5 shows the response to ethanol vapor/nitrogen mixture at ambient room temperature with 46% RH and a sensitivity of 1.15 ± 0.08. The sensitivity was obtained from the ratio between ambient resistance and resistance under analyte vapor (*R_a_*/*R_v_*), when ethanol vapor at 26.25 ppm is carried by nitrogen gas to the chemiresistor increasing the current through the thin film in as little as ∼0.6 s, so the resistance decrease quickly by 0.15% *i.e.*, applying Ohm’s Law (*R* = *V*/*I*) and normalized resistance change (ΔR/R_0_%), where R_0_ is the initial resistance in air and R the resistance measured in real-time. Furthermore, the sensing efficiency decrease only 1.26 % after 10 cycles (∼200 s).

In this chemiresistor, the MR dye molecule can be controlled by acid/base reactions. Consequently, the ions of the ethanol are transferred by MR to the PEDOT-PSS by adsorption in the diimide site, increasing the doped levels of the polymer blends and reducing the resistance. Similar process are related by Sadek or Krivan *et al.* [19,20]. In comparison with ZnO:Al thin film gas sensor [21], relative sensitivity for ethanol vapor/air mixture was ∼1.25 at 120 °C for 400 ppm concentration, but PEDOT-PSS doped with MR dye obtained responses at room temperature for detection limit of 26.25 ppm. The measures were realized applying DC bias of 2.50 V.

## 4. Conclusions

In conclusion, we have demonstrated a simple chemiresistor based on the PEDOT-PSS polymers doped with Methyl Red dye to form blend organic films. This gas sensor has been developed to monitor analyte with ethanol alcohol in real-time measurements.

The electrical characterizations shows reversible, quick and reproducible responses such as a limit detection of 26.25 ppm, sensitivity at 1.15 and a good response time of 0.6 s. The morphology of the thin film at 1,000 rpm is very favorable to increase adsorption of the analyte on the surface of the sensor element. This work indicates that doping conductor polymers with dye molecules is an alternative way to develop new chemiresistors.
